# Supplementary figures and images for: Similarities and differences: species and diet impact gut microbiota of captive pheasants
Source: PeerJ. 2024 Mar 26;12:e16979. doi: 10.7717/peerj.16979 (PMC10979745; doi:10.7717/peerj.16979)

**A**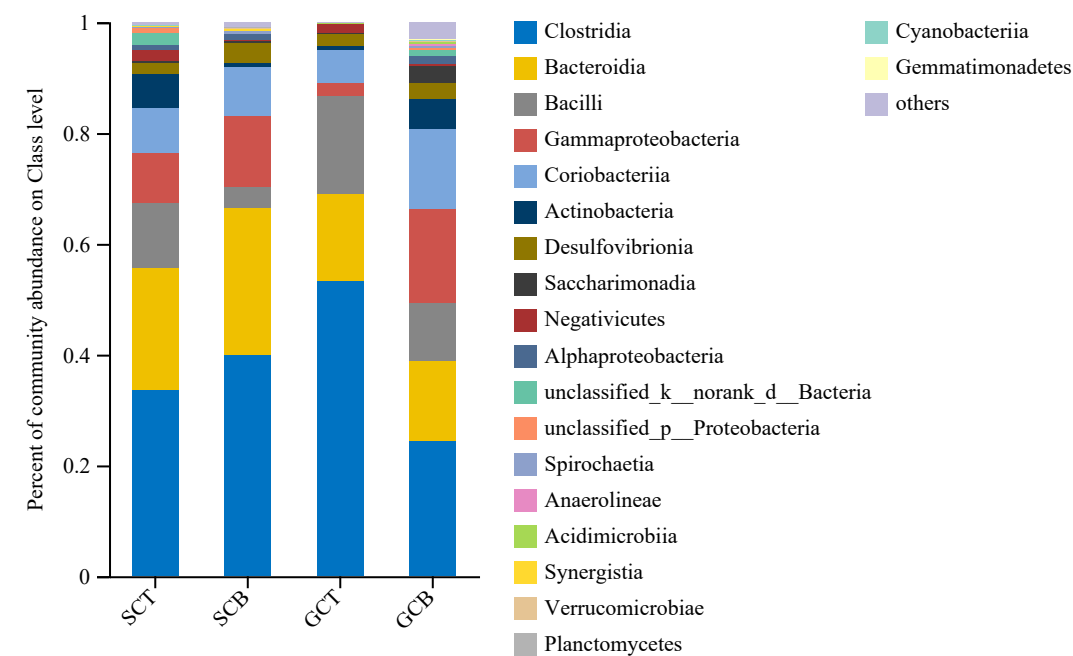**B**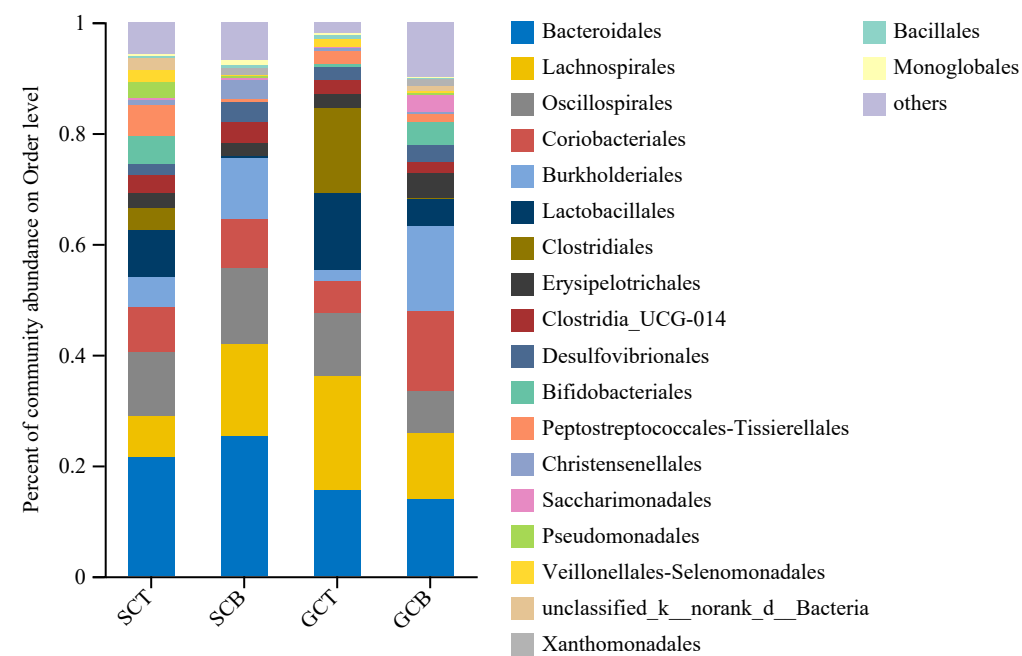**C**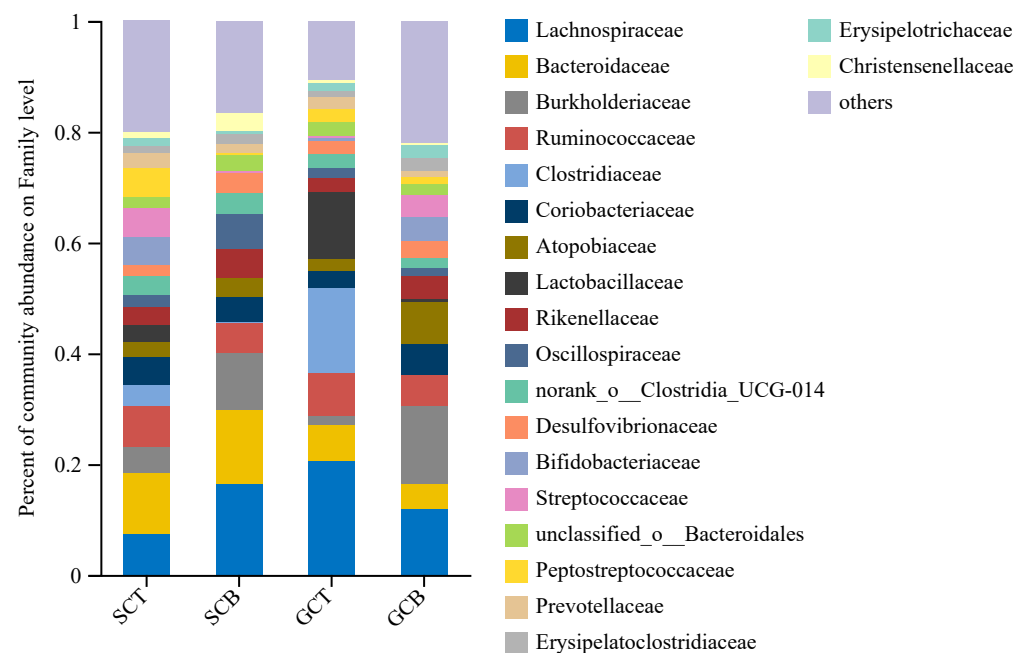

Supplement: Supplemental Information 2 [file peerj-12-16979-s002.pdf]

**A**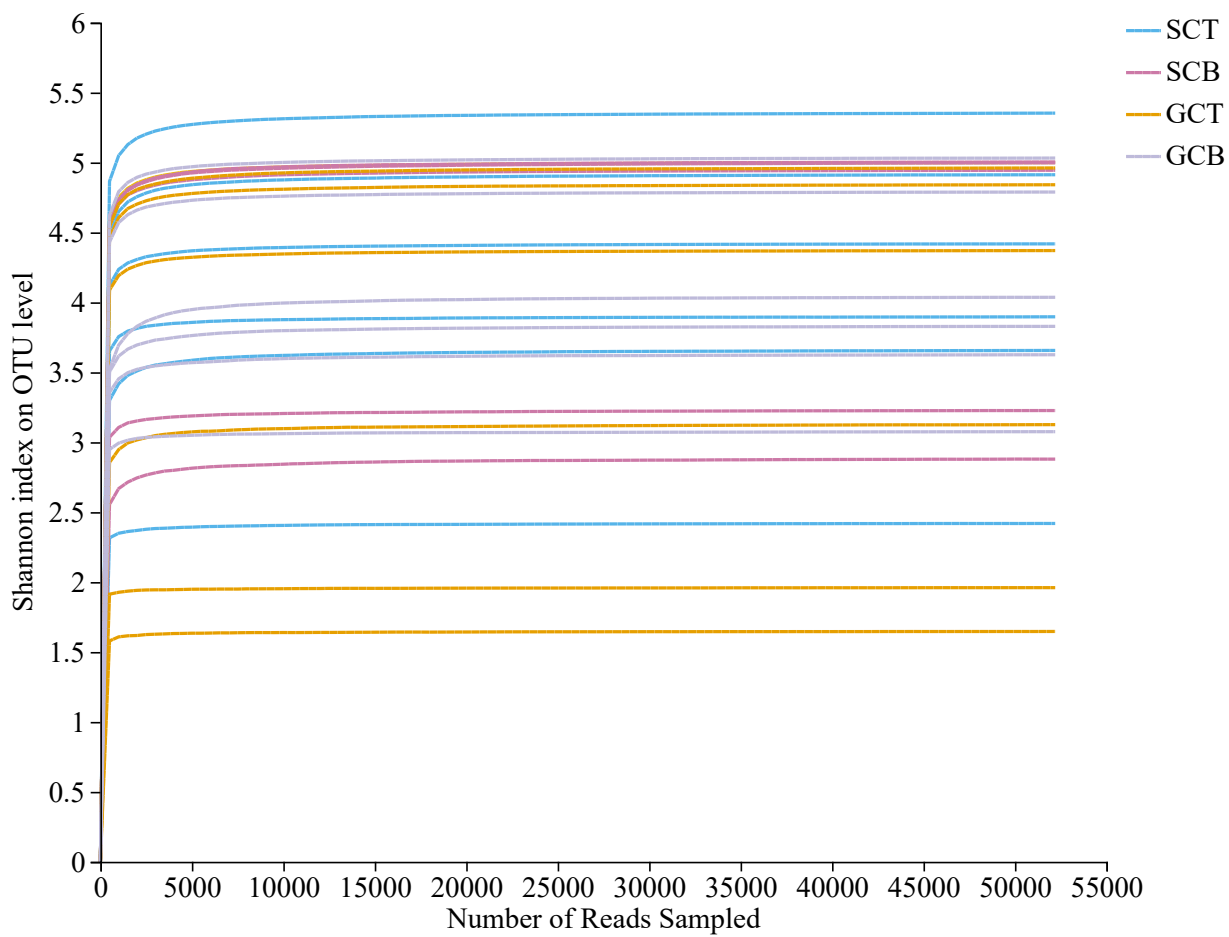**B**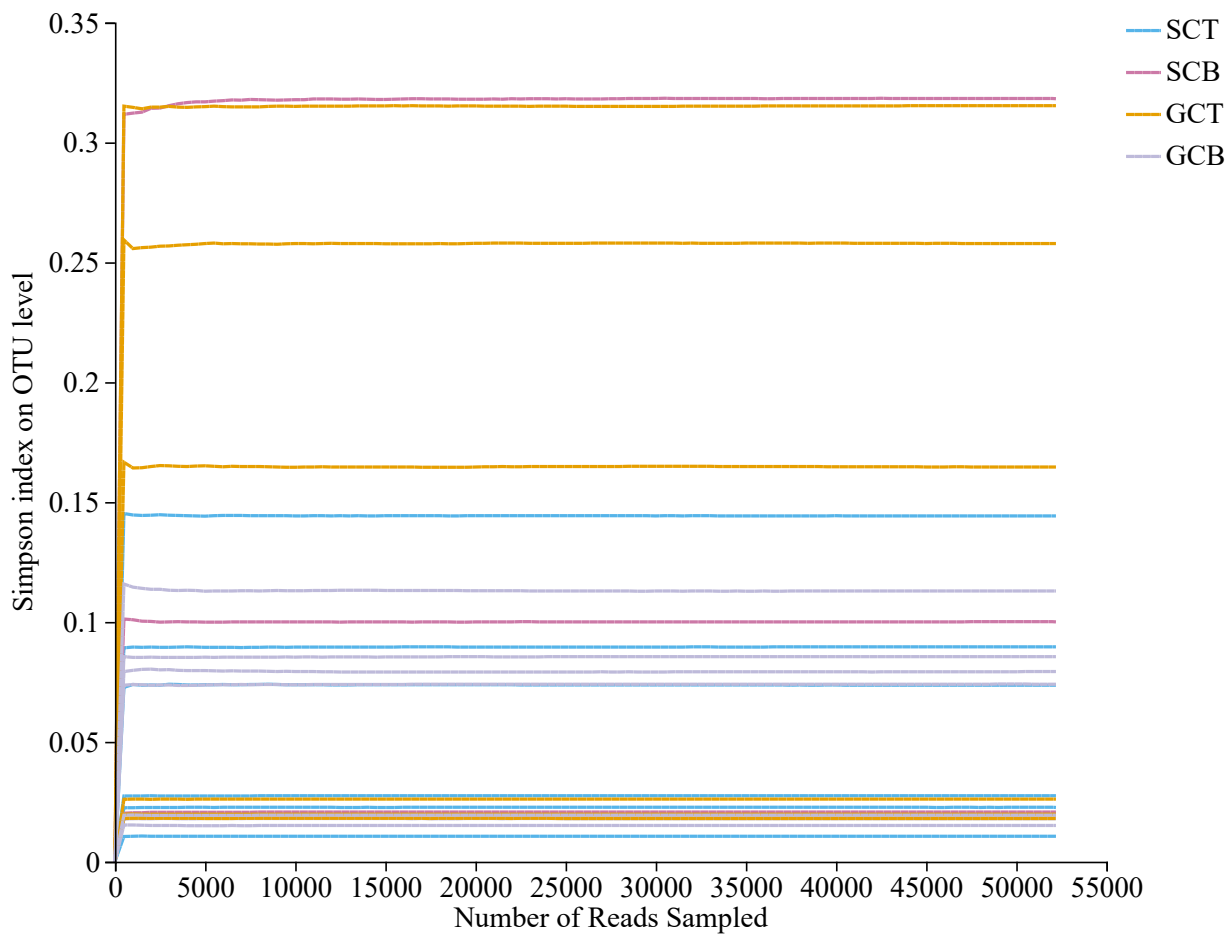

Supplement: Supplemental Information 3 [file peerj-12-16979-s003.pdf]

**A**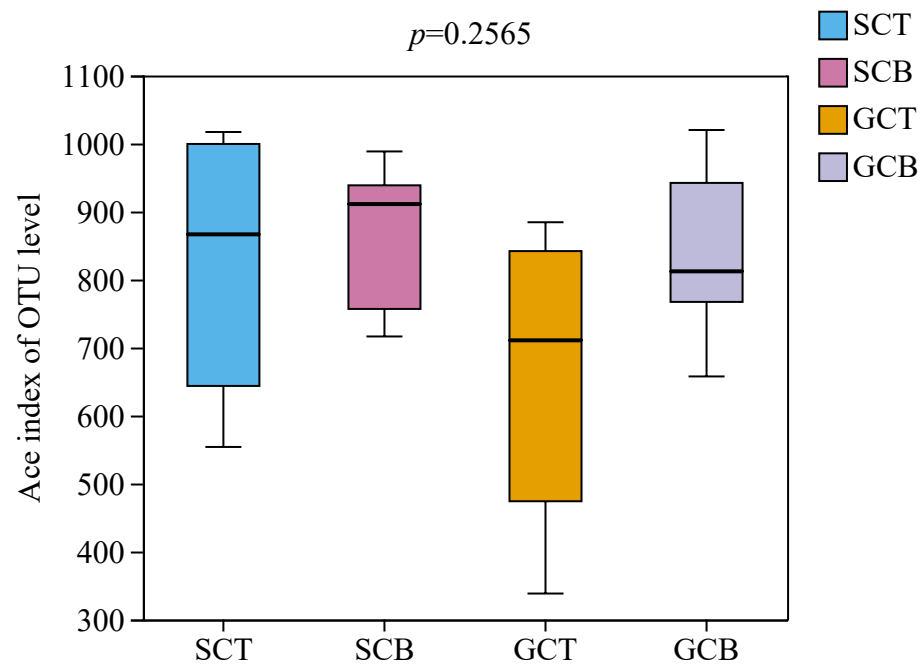**B**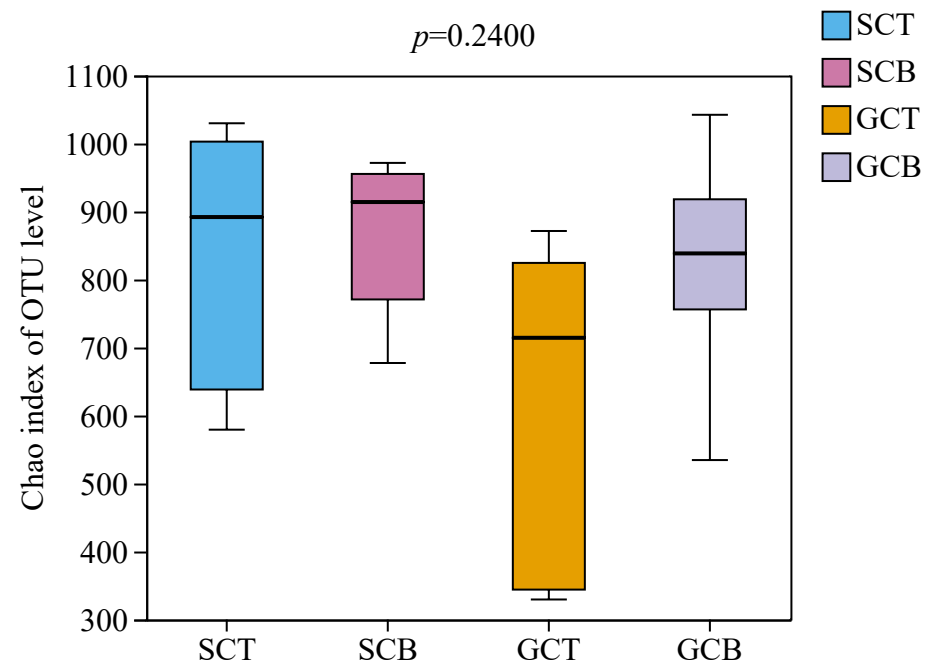**C**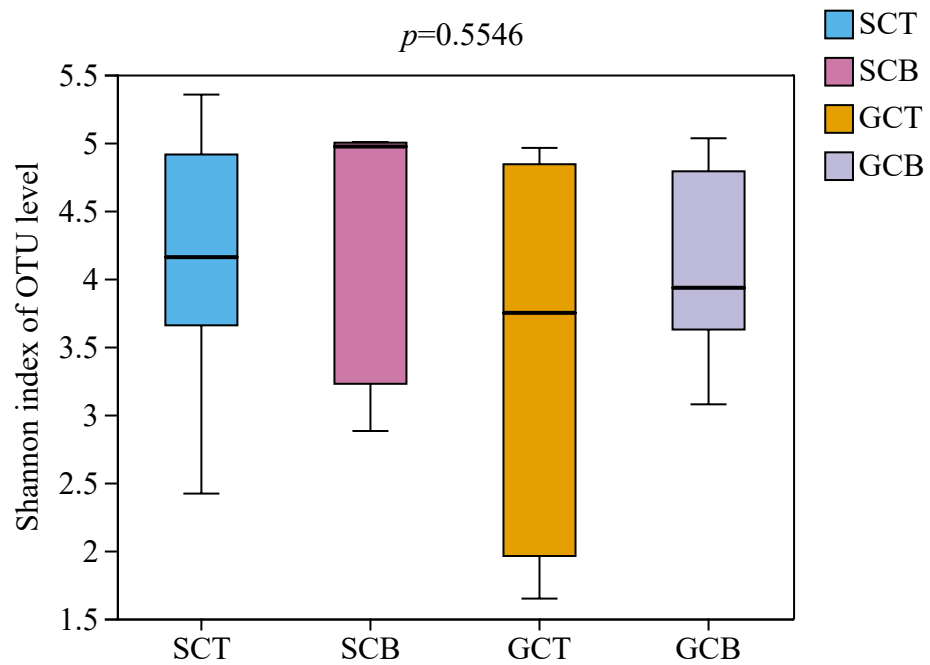**D**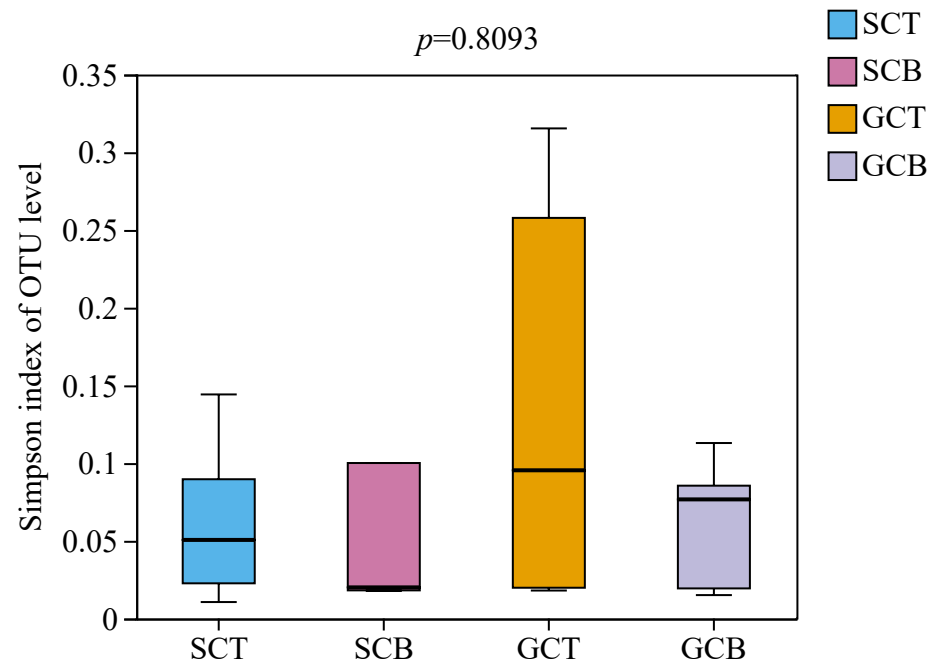

Supplement: Supplemental Information 4 — The alpha diversity index including Ace’s index (A), Chao’s index (B), Shannon’s index (C) and Simpson’s index (D) based on Student’s t-test at the OTU level. [file peerj-12-16979-s004.pdf]

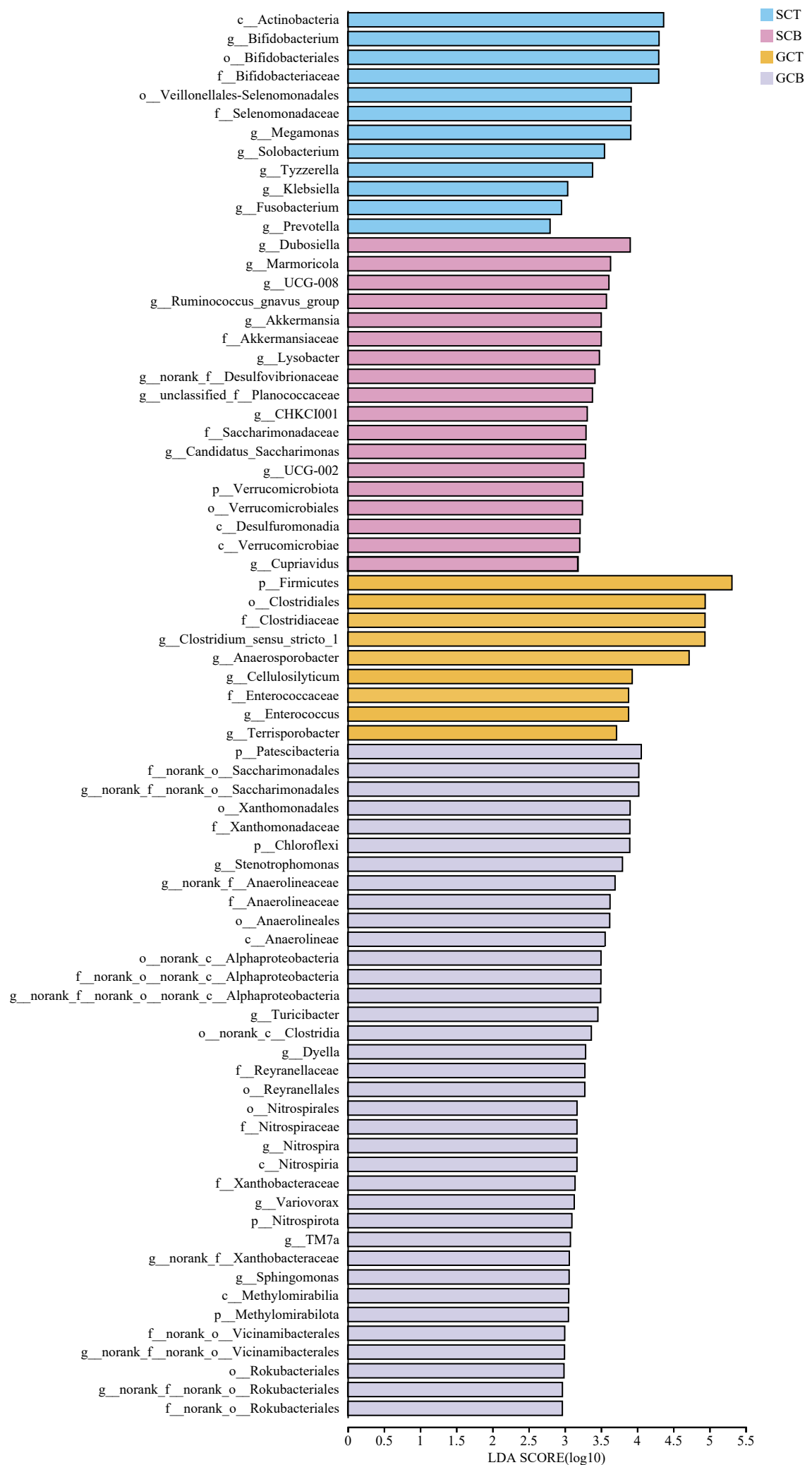

Supplement: Supplemental Information 5 — LDA scores indicated differences in abundance between the case and control groups (LDA scores > 2.0). [file peerj-12-16979-s005.pdf]

**A**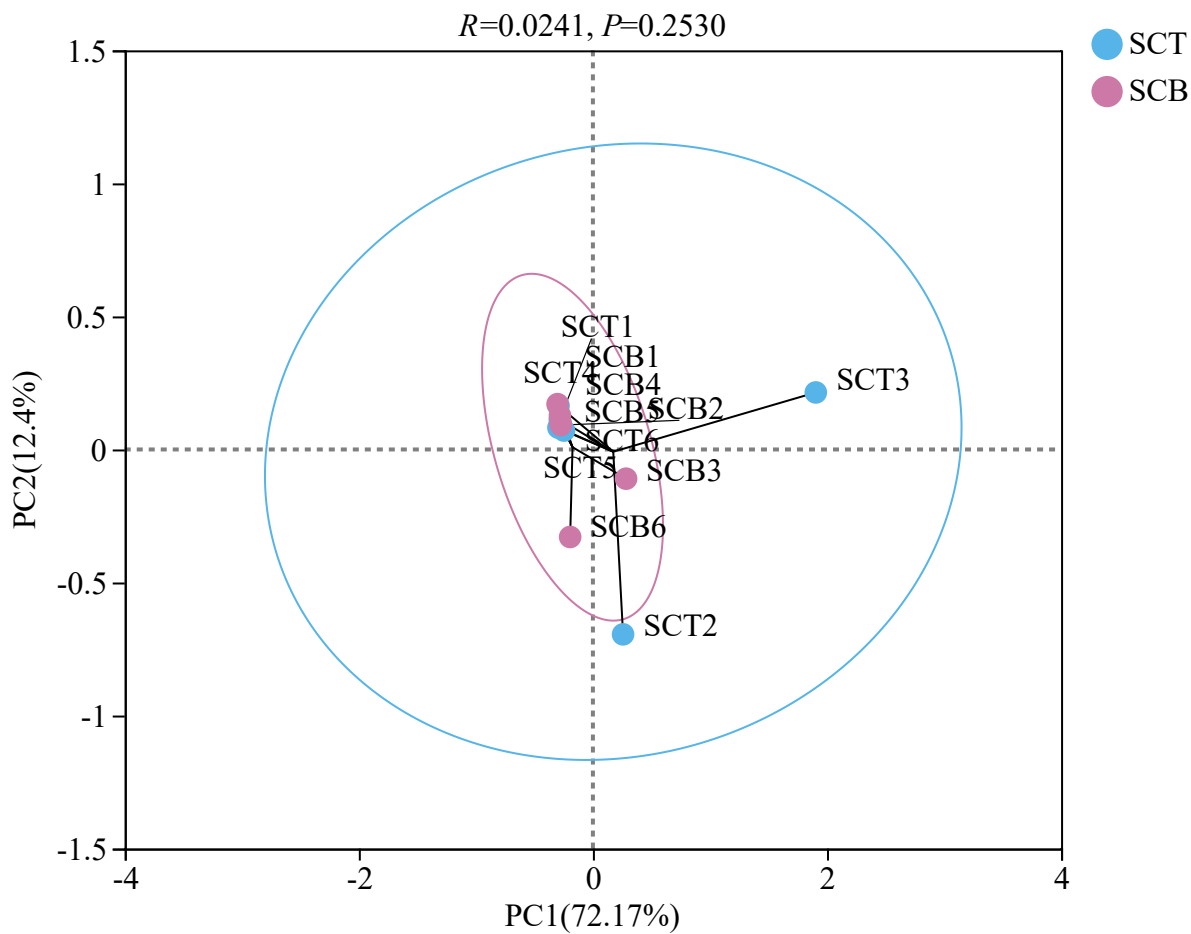**B**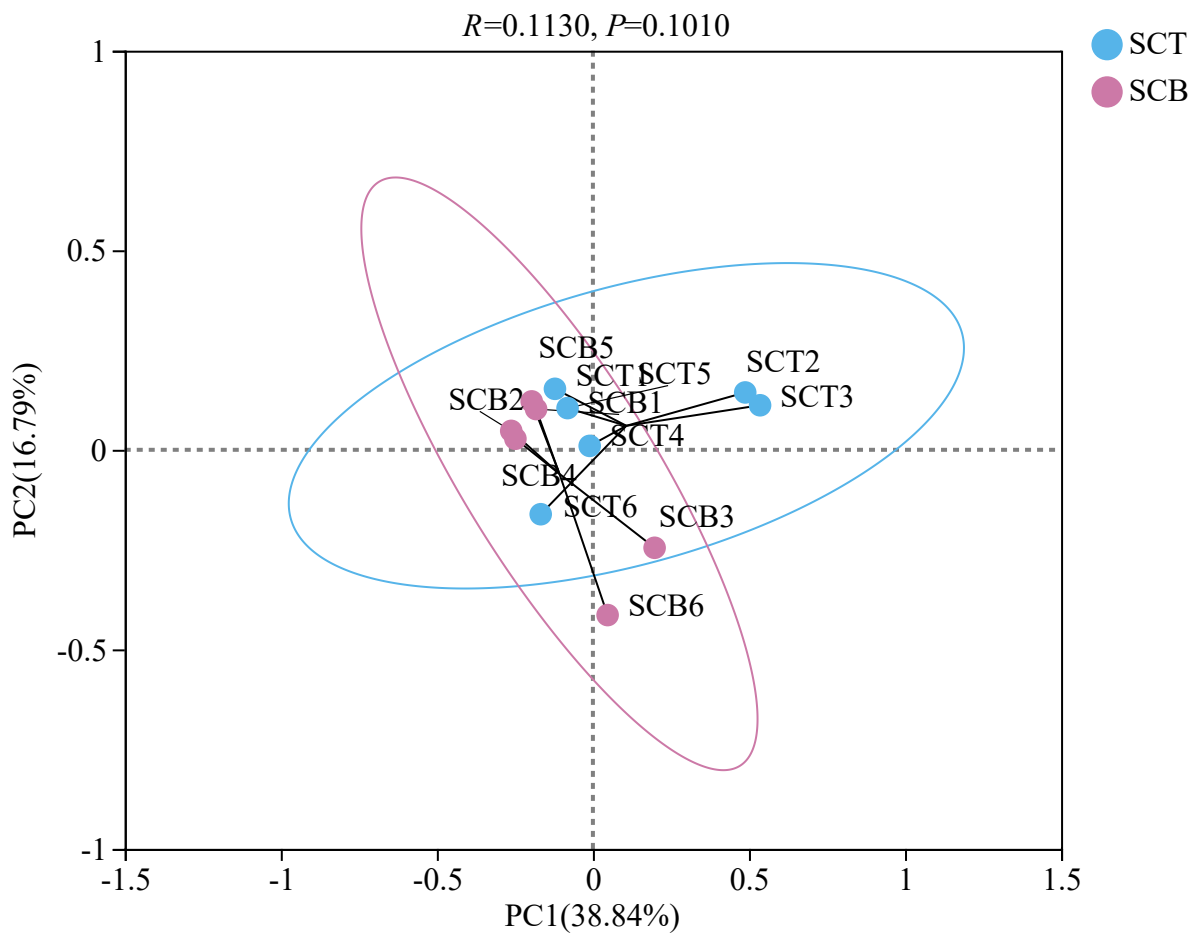

Supplement: Supplemental Information 6 [file peerj-12-16979-s006.pdf]

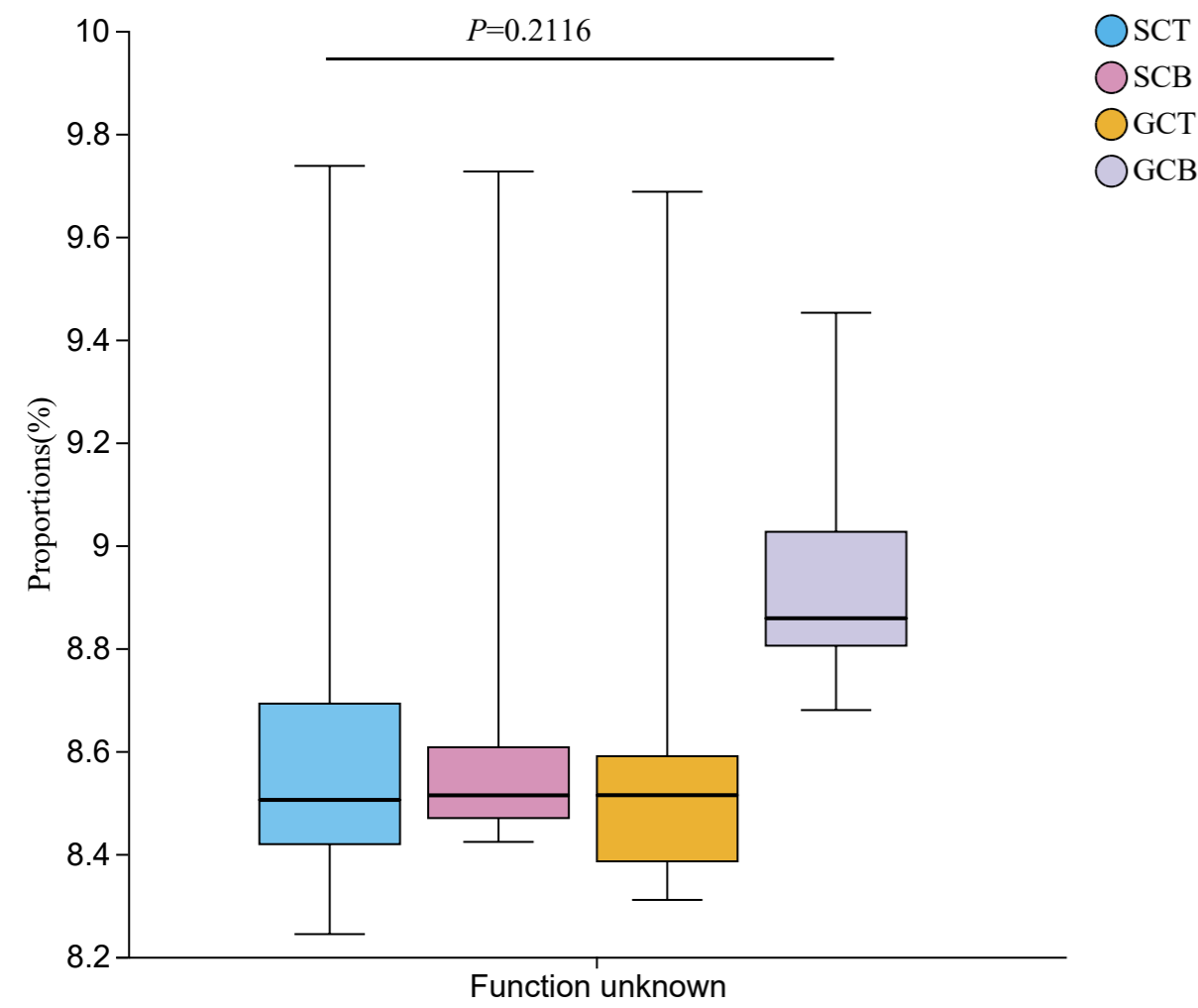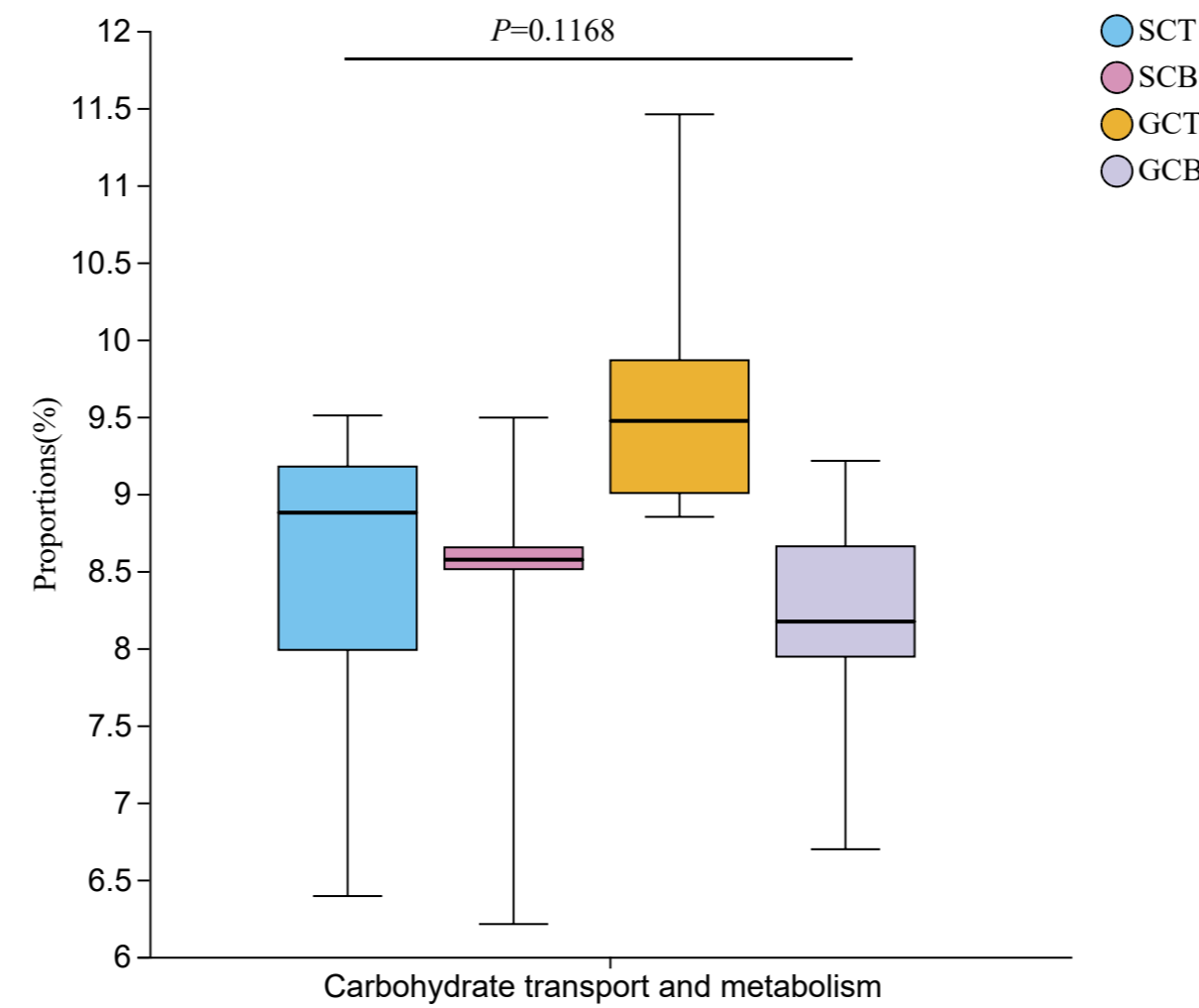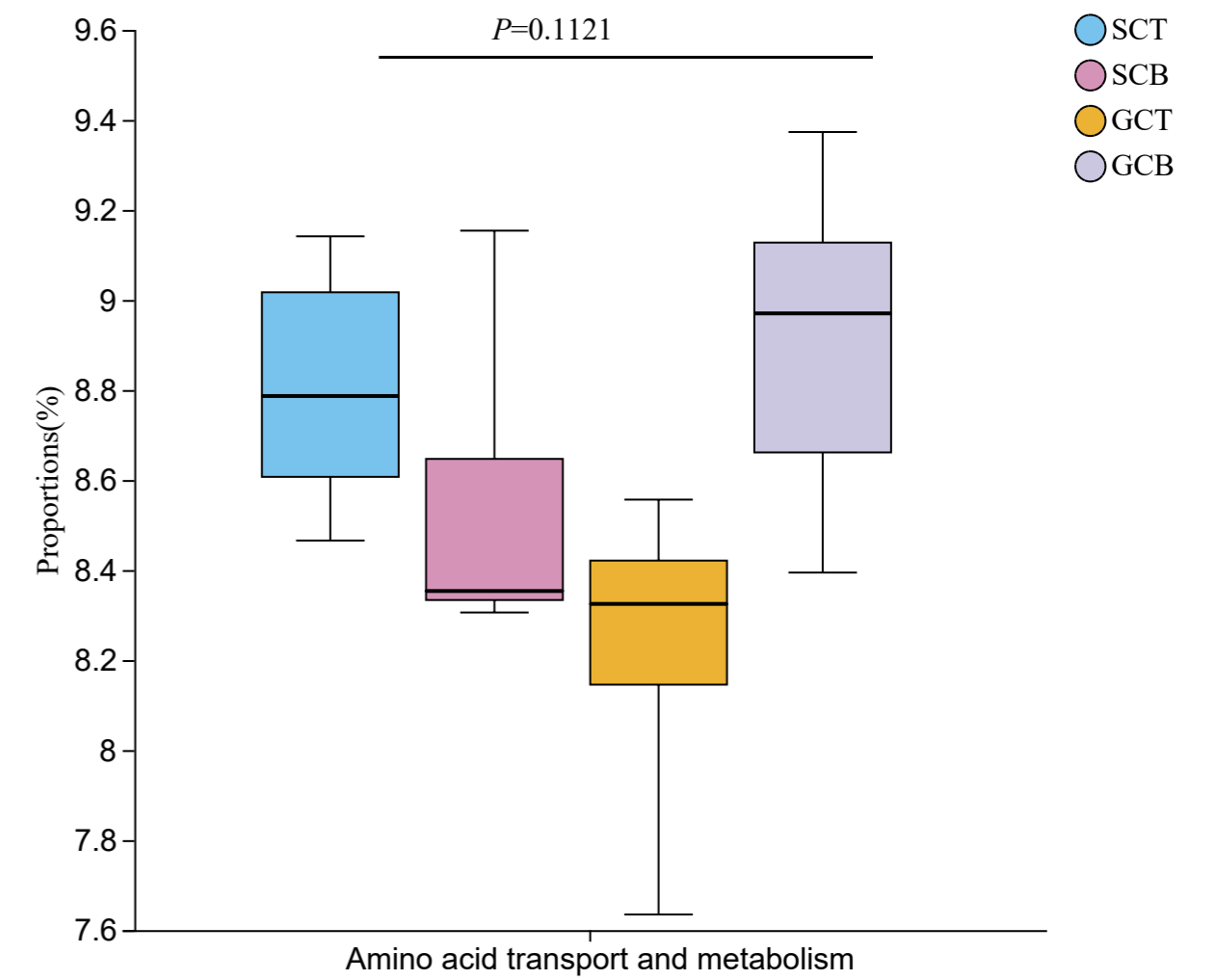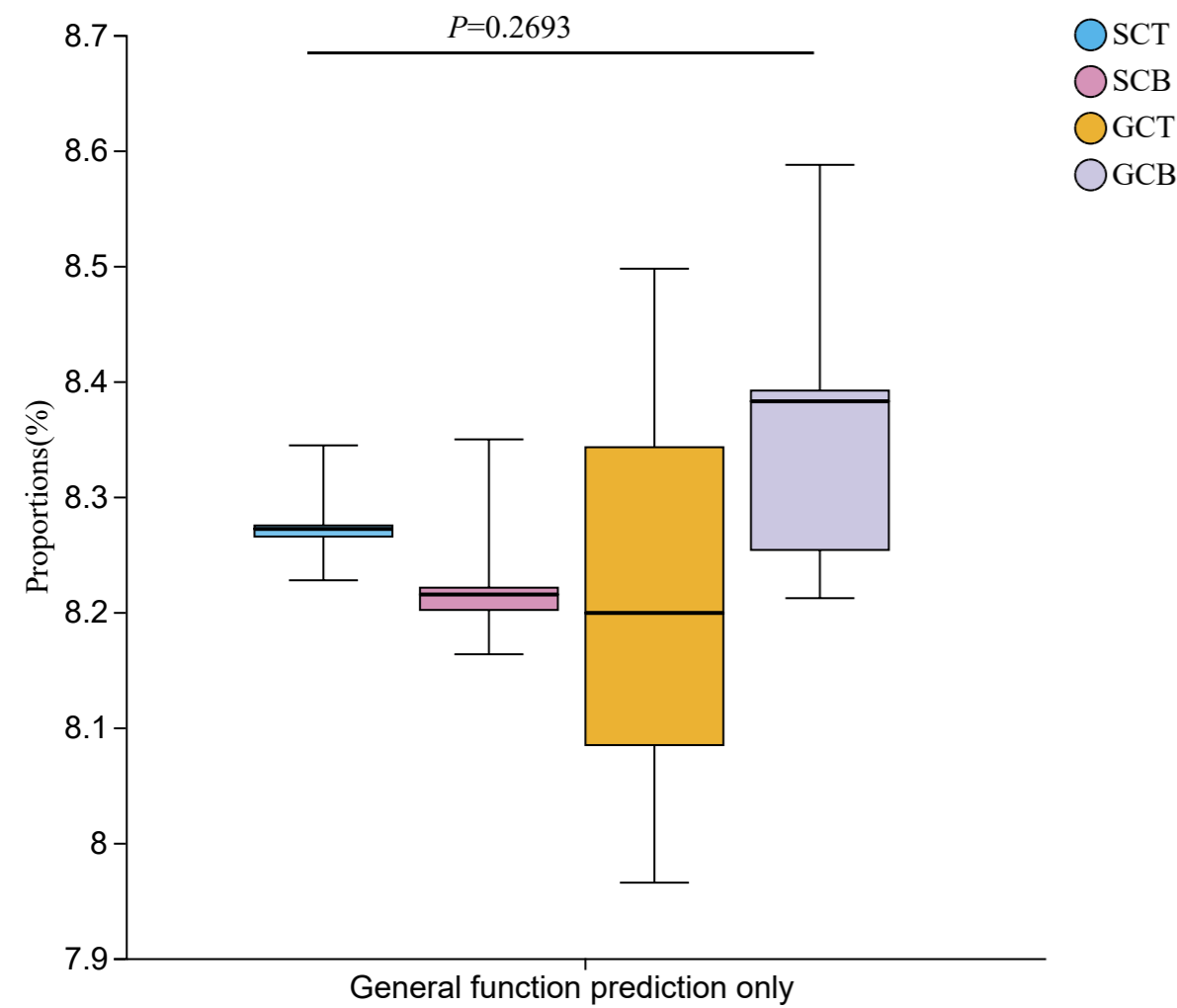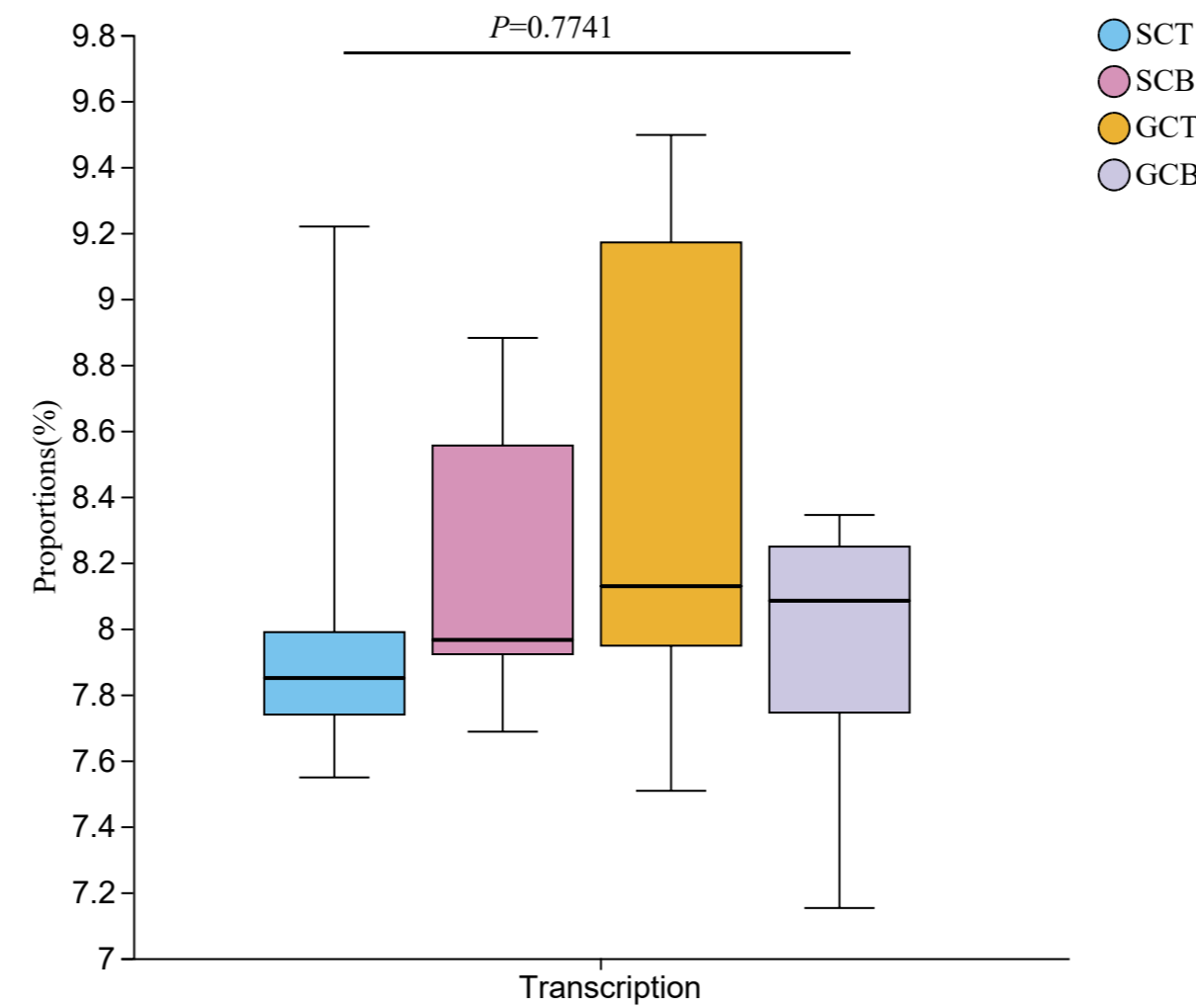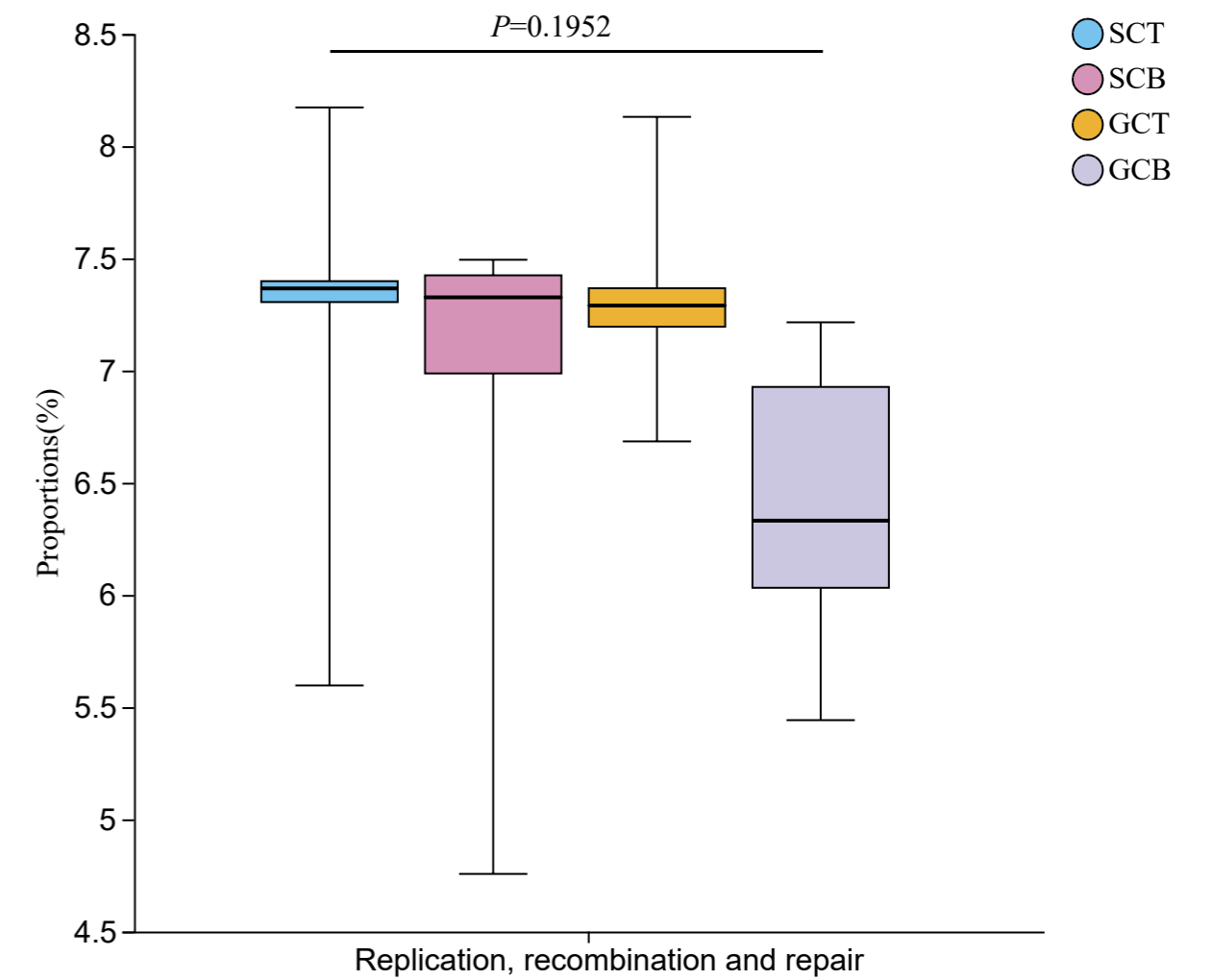

Supplement: Supplemental Information 7 [file peerj-12-16979-s007.pdf]

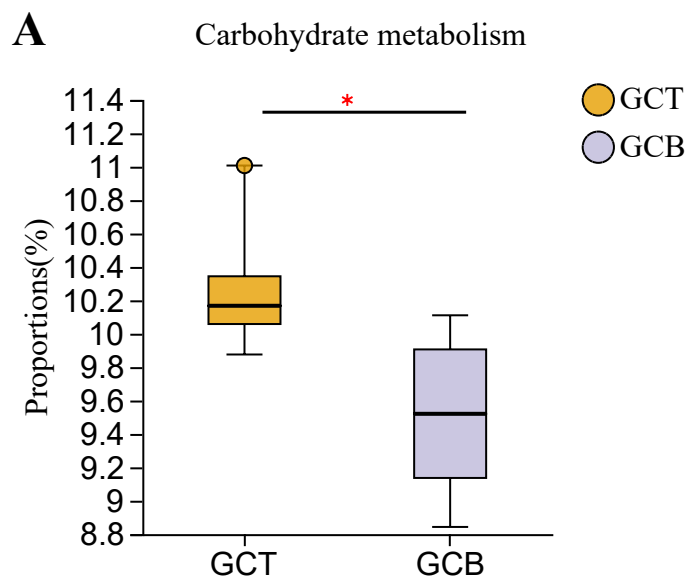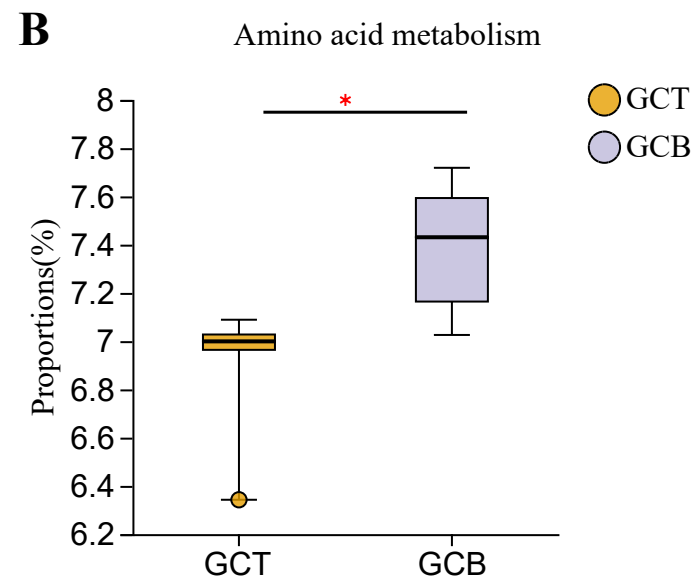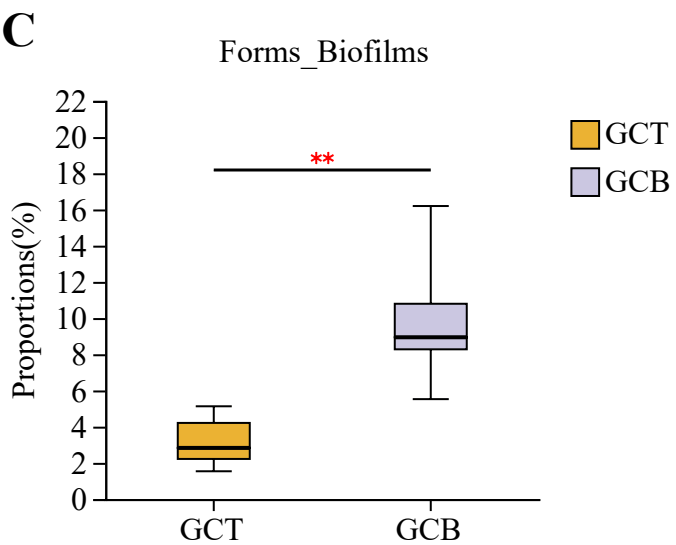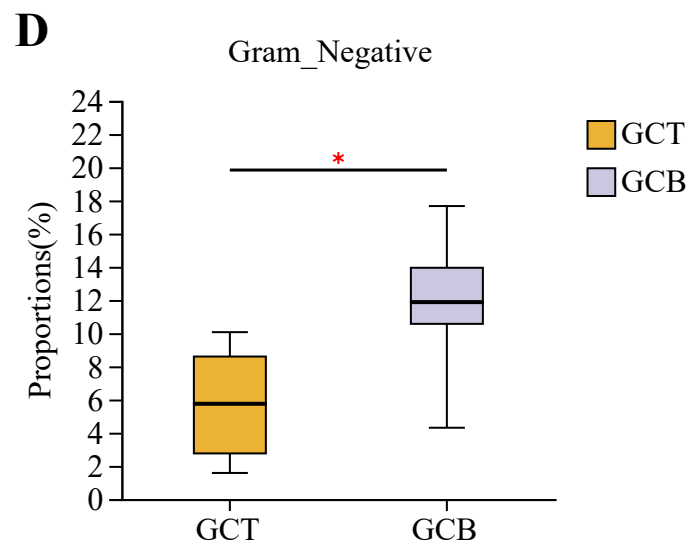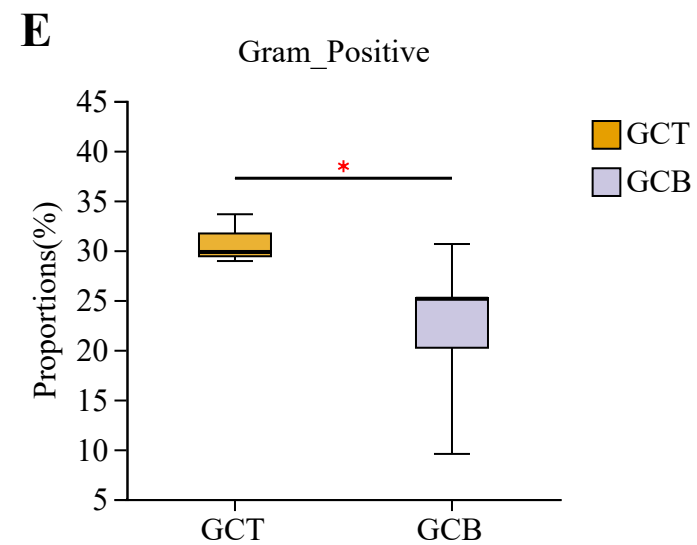

Supplement: Supplemental Information 8 — Carbohydrate metabolism (A) and amino acid metabolism (B) pathways were significantly different between GCB and GCT; Biofilm Forming phenotypes (C), Gram-positive phenotypes (D) and Gram-negative phenotypes (E) were significantly different between GCB and GCT. Significant difference is represented by * P ≤ 0.05. [file peerj-12-16979-s008.pdf]
