# Supplementary material for: Similarities and differences: species and diet impact gut microbiota of captive pheasants
Source: PeerJ. 2024 Mar 26;12:e16979. doi: 10.7717/peerj.16979 (PMC10979745; doi:10.7717/peerj.16979)
Supplement: Supplemental Information 9 [file peerj-12-16979-s009.docx]

**Table S1.** The mean length of the amplified sequence from all fecal samples.

| **Sample** | **Mean_length** | **Min_length** | **Max_length** |
| --- | --- | --- | --- |
| SCT1 | 412.511409 | 248 | 450 |
| SCT2 | 409.693187 | 213 | 449 |
| SCT3 | 395.987872 | 217 | 449 |
| SCT4 | 414.756805 | 247 | 450 |
| SCT5 | 411.893405 | 248 | 445 |
| SCT6 | 410.213265 | 115 | 452 |
| SCB1 | 414.397938 | 239 | 452 |
| SCB2 | 412.237298 | 257 | 450 |
| SCB3 | 422.313304 | 231 | 452 |
| SCB4 | 413.923543 | 213 | 452 |
| SCB5 | 412.332581 | 232 | 449 |
| SCB6 | 405.539541 | 222 | 450 |
| GCT1 | 422.16857 | 231 | 428 |
| GCT2 | 405.842015 | 230 | 429 |
| GCT3 | 411.722331 | 179 | 451 |
| GCT4 | 411.204434 | 291 | 451 |
| GCT5 | 412.340815 | 302 | 449 |
| GCT6 | 405.836885 | 197 | 451 |
| GCB1 | 416.042947 | 271 | 452 |
| GCB2 | 410.285976 | 212 | 452 |
| GCB3 | 407.513301 | 213 | 449 |
| GCB4 | 409.689956 | 100 | 447 |
| GCB5 | 414.976126 | 195 | 452 |
| GCB6 | 415.40154 | 100 | 447 |
