# Supplementary material for: Similarities and differences: species and diet impact gut microbiota of captive pheasants
Source: PeerJ. 2024 Mar 26;12:e16979. doi: 10.7717/peerj.16979 (PMC10979745; doi:10.7717/peerj.16979)
Supplement: Supplemental Information 10 [file peerj-12-16979-s010.docx]

**Table S2.** Estimated OTU richness and diversity indexes for each fecal sample.

| Sample ID | ACE | Chao | Shannon | Simpson | Coverage |
| --- | --- | --- | --- | --- | --- |
| SCT1 | 904 | 1016.946835 | 1029.783133 | 5.352469 | 0.0106 |
| SCT2 | 375 | 553.781731 | 579.185185 | 2.418665 | 0.144176 |
| SCT3 | 777 | 950.160179 | 993.723684 | 3.655836 | 0.089613 |
| SCT4 | 645 | 782.706293 | 790 | 4.41777 | 0.027516 |
| SCT5 | 874 | 999.3501 | 1002.8 | 4.911961 | 0.022649 |
| SCT6 | 511 | 643.512792 | 638.258621 | 3.896348 | 0.07362 |
| SCB1 | 788 | 887.002977 | 893.558442 | 4.998979 | 0.019388 |
| SCB2 | 850 | 934.907437 | 955.276316 | 5.003558 | 0.020683 |
| SCB3 | 680 | 756.749944 | 770.71831 | 2.878786 | 0.31823 |
| SCB4 | 806 | 938.523614 | 934.333333 | 4.944095 | 0.017919 |
| SCB5 | 853 | 988.359036 | 971.5 | 4.996964 | 0.018113 |
| SCB6 | 518 | 716.262796 | 677.244186 | 3.225899 | 0.10005 |
| GCT1 | 651 | 754.435409 | 766.727273 | 3.125319 | 0.164607 |
| GCT2 | 228 | 338.216559 | 329.477273 | 1.646592 | 0.315334 |
| GCT3 | 788 | 884.240261 | 871.366667 | 4.960437 | 0.018008 |
| GCT4 | 745 | 841.801125 | 824.4375 | 4.840437 | 0.019806 |
| GCT5 | 563 | 666.701092 | 661.875 | 4.369851 | 0.026124 |
| GCT6 | 183 | 473.922971 | 344 | 1.959706 | 0.257755 |
| GCB1 | 694 | 767.071572 | 756.045455 | 4.788333 | 0.019375 |
| GCB2 | 344 | 657.474795 | 534.5 | 3.074927 | 0.085471 |
| GCB3 | 665 | 780.050264 | 799.638554 | 3.624907 | 0.079276 |
| GCB4 | 750 | 844.018042 | 877.147059 | 3.827916 | 0.074019 |
| GCB5 | 788 | 942.086159 | 918 | 5.031343 | 0.015072 |
| GCB6 | 963 | 1019.868181 | 1042.246154 | 4.035845 | 0.112914 |
