# Supplementary material for: Similarities and differences: species and diet impact gut microbiota of captive pheasants
Source: PeerJ. 2024 Mar 26;12:e16979. doi: 10.7717/peerj.16979 (PMC10979745; doi:10.7717/peerj.16979)
Supplement: Supplemental Information 1 [file peerj-12-16979-s001.pdf]

A

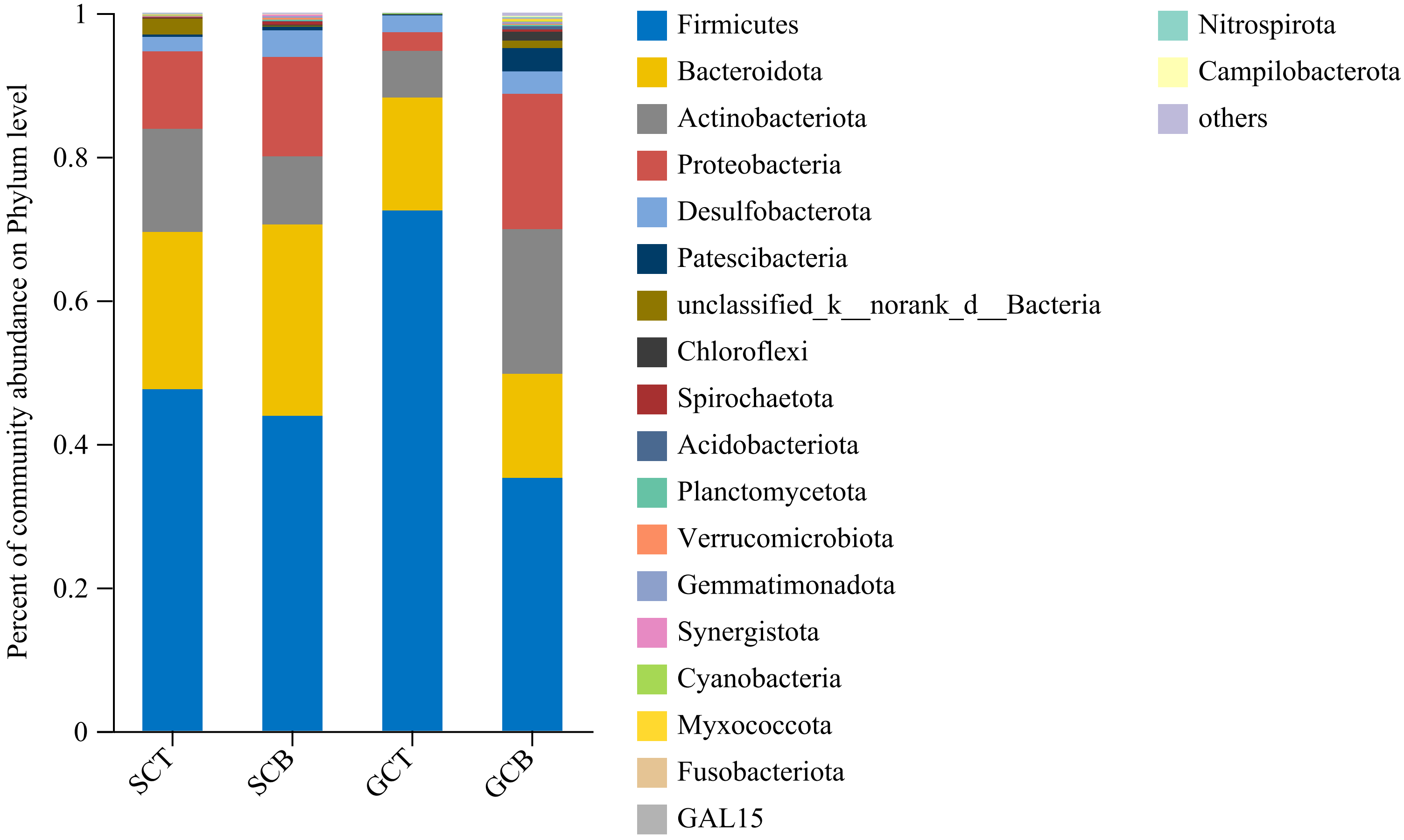

Percent of community abundance on Phylum level
1
0.8
0.6
0.4
0.2
0
SCT
SCB
GCT
GCB
Firmicutes
Bacteroidota
Actinobacteriota
Proteobacteria
Desulfobacterota
Patescibacteria
unclassified_k__norank_d__Bacteria
Chloroflexi
Spirochaetota
Acidobacteriota
Planctomycetota
Verrucomicrobiota
Gemmatimonadota
Synergistota
Cyanobacteria
Myxococcota
Fusobacteriota
GAL15
Nitrospirota
Campilobacterota
others

B

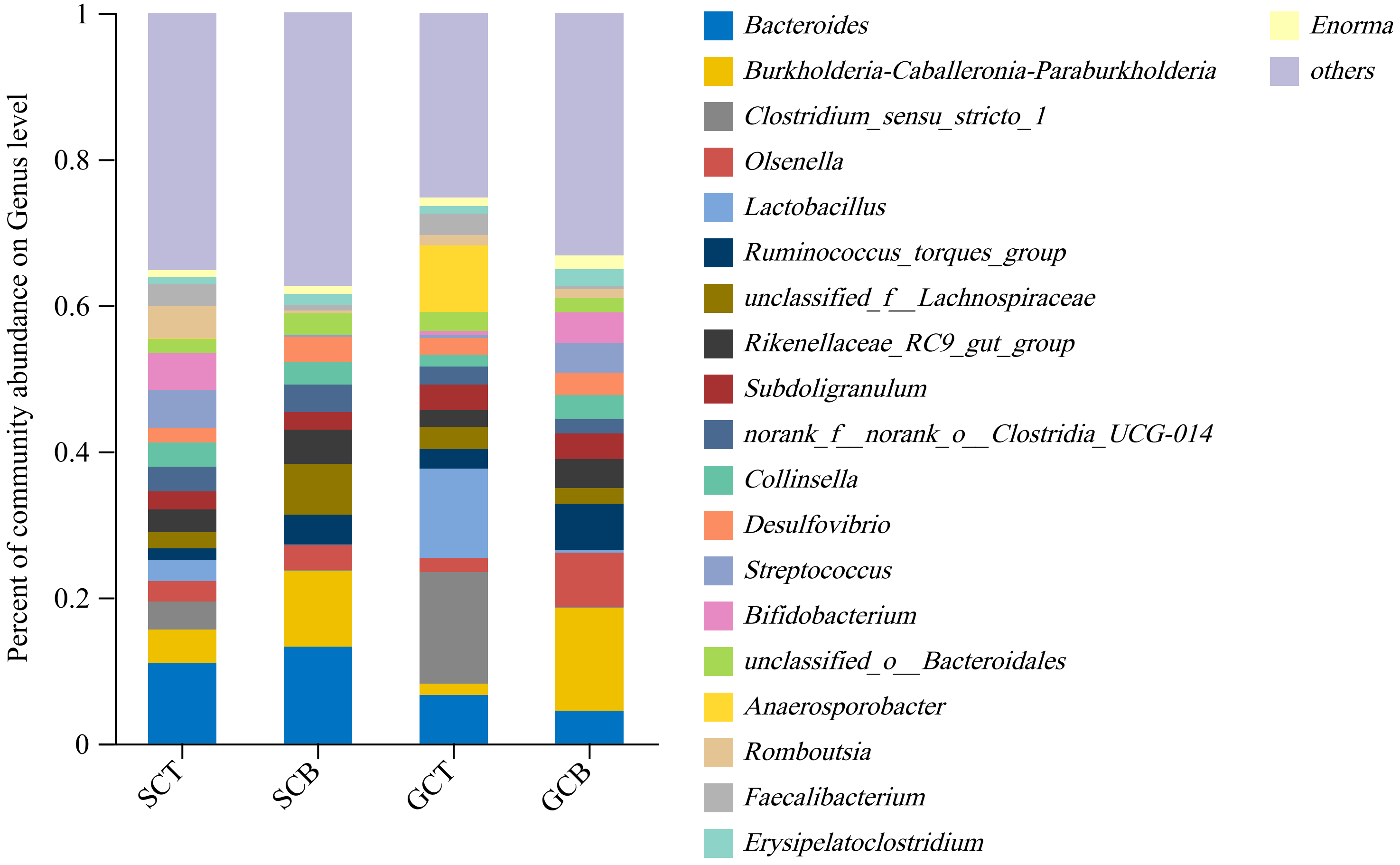

Percent of community abundance on Genus level
1
0.8
0.6
0.4
0.2
0
SCT
SCB
GCT
GCB
Bacteroides
Burkholderia-Caballeronia-Paraburkholderia
Clostridium_sensu_stricto_1
Olsenella
Lactobacillus
Ruminococcus_torques_group
unclassified_f__Lachnospiraceae
Rikenellaceae_RC9_gut_group
Subdoligranulum
norank_f__norank_o__Clostridia_UCG-014
Collinsella
Desulfovibrio
Streptococcus
Bifidobacterium
unclassified_o__Bacteroidales
Anaerosporobacter
Romboutsia
Faecalibacterium
Erysipelatoclostridium
Enorma
others
